# Prevalence and risk factors of hypertension in Saudi Arabia based on a nationally representative survey

**DOI:** 10.3389/fcvm.2026.1752357

**Published:** 2026-02-10

**Authors:** Najim Z. Alshahrani, Abdullah M. Alarifi, Abdulghaffar H. Humieda, Shaker A. Alomary, Manal A. Elimam, Mohammed S. Aldossary, Arwa M. Alshangiti, Sultan S. Alamri, Ahmed K. Shukri, Wejdan J. Aloufi, Saeed H. Alqahtani, Yahya Al Asseri, Abdullah M. Assiri

**Affiliations:** 1 Department of Family and Community Medicine, Faculty of Medicine, University of Jeddah, Jeddah, Saudi Arabia; 2 Deputyship of Population Health, Ministry of Health, Riyadh, Saudi Arabia; 3 General Directorate of Statistics and Information, Ministry of Health, Riyadh, Saudi Arabia; 4 Department of Health Programs and Chronic Diseases, Deputyship of Population Health, Ministry of Health, Riyadh, Saudi Arabia; 5 General Directorate of Research and Studies, Ministry of Health, Riyadh, Saudi Arabia; 6 Department of Statistics and Operations Research, College of Science, King Saud University, Riyadh, Saudi Arabia

**Keywords:** chronic diseases, hypertension, HTN, prevalence, risk factors, Saudi Arabia

## Abstract

**Background:**

Hypertension is a leading cause of cardiovascular diseases, stroke, and kidney failure worldwide. Despite being a preventable and manageable condition, hypertension often remains undiagnosed or poorly controlled, contributing to substantial health burdens. This study aims to estimate the prevalence of hypertension among adults in Saudi Arabia and identify key sociodemographic and behavioral risk factors associated with the condition.

**Methods:**

This study utilized data from the 2019 Kingdom of Saudi Arabia World Health Survey (KSAWHS 2019), a nationally representative survey conducted by the Ministry of Health. The original sample included 8,912 individuals aged 15 years and older. After data cleaning, the analytical sample consisted of 8,618 individuals in this age group. For the present analysis, a subset of 8,266 adults aged 18 years and older with valid blood pressure measurements was used. Hypertension prevalence was estimated as both crude and age-standardized rates, with standardization based on the WHO 2000–2025 standard population. Poisson regression with robust variance was employed to assess associations between hypertension and a range of demographic, clinical, behavioral, and biochemical factors.

**Results:**

Among adults aged 18 years and older, the crude prevalence of hypertension was 12.69%. Hypertension prevalence increased markedly with age, reaching over 50% among adults aged 80 years and older. Males had a higher prevalence of hypertension than females (14.1% vs. 11.2%). Among adults aged 30–79 years, the crude prevalence of hypertension was 19.41%, with 47.96% of affected individuals receiving antihypertensive treatment; the corresponding age-standardized prevalence and treatment coverage were 22.26% and 43.97%, respectively. In multivariable analyses, hypertension was significantly associated with older age, kidney disease (PR = 2.05), obesity (PR = 1.72), dyslipidemia (PR = 1.57), diabetes (PR = 1.34), and ever smoking (PR = 1.19). Among adults with hypertension, only 34.8% were aware of their condition.

**Conclusion:**

Hypertension remains a major public health challenge in Saudi Arabia. Additionally, hypertension awareness in Saudi Arabia is critically low, representing the most important gap in hypertension control. Key risk factors such as obesity, smoking, and diabetes significantly contribute to the burden of the disease. Strengthening routine blood pressure screening, expanding community-based detection initiatives, and integrating hypertension awareness into national programs are essential to improve early diagnosis and long-term cardiovascular outcomes.

## Introduction

Saudi Arabia's population was estimated at 35.3 million by mid−2024, with approximately 37% of them under the age of 25. The country is divided into 13 administrative regions, covering an area of approximately 2,150,000 square kilometers. In recent years, shifts in lifestyle and dietary habits have contributed to a rising burden of noncommunicable diseases, such as obesity, diabetes, and cardiovascular diseases (1). One of the most common of these is hypertension (2). Hypertension is a major public health issue worldwide, including in Saudi Arabia (3). It is a leading risk factor for cardiovascular disease, stroke, and kidney failure (3). Despite being a preventable and manageable condition, hypertension is the second most common cause of avoidable death after smoking (3). Because it often has no clear symptoms, it can go undiagnosed or remain poorly controlled.

Several factors increase the risk of hypertension. Age and male gender are significant non-modifiable risk factors (4). Social and economic factors also play a role in individuals with fewer years of education and lower income levels tend to be at higher risk (5, 6). In addition, overweight and obesity are strongly linked to high blood pressure, while individuals who are underweight tend to have a lower risk (7). Conditions such as diabetes mellitus, dyslipidemia, and low levels of high-density lipoprotein cholesterol further contribute to the development of hypertension (8).

Unhealthy diets, particularly those high in salt and fat, combined with low intake of fruits and vegetables, are also associated with higher rates of hypertension (9). Frequent consumption of fast food, physical inactivity, and high levels of stress have been shown to worsen the problem (10). As a result, hypertension has become a leading cause of health-related economic loss in many countries (11).

Effective management of hypertension can reduce the risk of complications and ease the burden on the healthcare system (10). However, many cases remain untreated or poorly controlled. While global efforts have aimed to improve hypertension awareness and treatment, progress has been uneven across countries (12). Differences in age distribution, healthcare access, and education may explain the variation in prevalence and control rates (12). Research shows that lowering systolic blood pressure by 10 mmHg can reduce cardiovascular risk by up to 30%. Early detection and treatment are essential to prevent serious outcomes and reduce healthcare costs (13).

In Saudi Arabia, several studies have investigated blood pressure trends over the last three decades. A recent systematic review and meta-analysis conducted in 2023 found that the prevalence of hypertension ranged from 15.2% to 32.6% in national studies, and from 4.2% to 71.3% in regional studies (3). According to a national study published in 2023, the overall prevalence of hypertension among the Saudi population aged 15 years and older was 9.2% (11). The condition was slightly more common in women (10%) compared to men (8.5%).

Given the wide variation in hypertension prevalence across different regions of Saudi Arabia and the growing concern over its public health impact, it is important to update and expand the understanding of this condition at the national level. This study aims to estimate the prevalence of hypertension among adults in Saudi Arabia and to identify the key associated sociodemographic and behavioral risk factors.

## Materials and methods

### Study design and data source

This cross-sectional study utilized data from the Kingdom of Saudi Arabia World Health Survey 2019 (KSAWHS 2019), a nationally representative household survey conducted by the Ministry of Health (MoH) (14, 15). The survey was designed to produce reliable and up-to-date estimates of health indicators aligned with Sustainable Development Goals (SDGs), the WHO Global Reference List of 100 Core Health Indicators, and national health priorities (16). A stratified three-stage cluster sampling design was employed across all 13 administrative regions of Saudi Arabia, covering both urban and rural areas. In the first stage, enumeration areas (EAs) were selected with probability proportional to size using the updated 2010 Saudi Population and Housing Census as the sampling frame. In the second stage, eight households were systematically selected from each EA. In the third stage, one individual aged 15 years or older was randomly selected from each household to participate in the individual interview and biometric measurements.

Survey weights were calculated to account for differential probabilities of selection and non-response, and were normalized to ensure national representativeness. All participants provided written informed consent. Ethical approval for the survey was granted by the General Directorate of Research and Studies at the Ministry of Health.

### Study population and analytic samples

**Figure 1:**
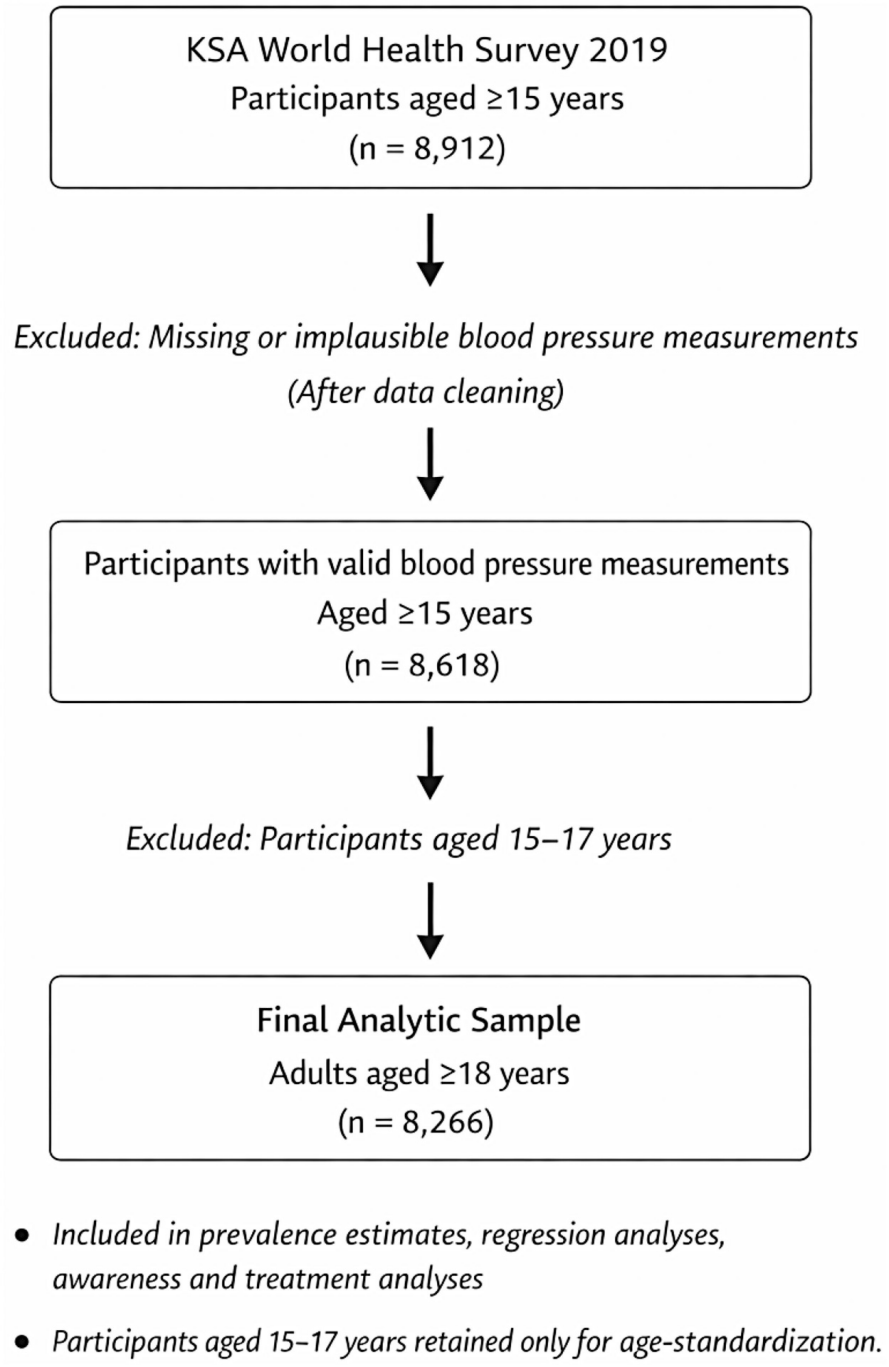
Flow diagram of study population selection.

The original survey sample consisted of 8,912 participants aged 15 years and older who completed the interview and blood pressure measurements. A structured data-cleaning protocol was applied to blood pressure measurements to address missing and implausible values. After exclusion of observations with missing or physiologically implausible blood pressure readings, 8,618 participants aged ≥15 years remained with valid blood pressure data. The process of study population selection and exclusions is illustrated in Figure 1.

For the primary analyses of hypertension prevalence, risk factors, awareness, and treatment, the analytic sample was restricted to 8,266 adults aged ≥18 years, reflecting the adult population and ensuring consistency across descriptive and multivariable analyses. Participants aged 15–17 years were excluded from all descriptive and regression analyses, but were retained exclusively for age-standardization. This approach was adopted to align with the WHO World Standard Population (2000–2025), which includes the 15–19 year age group, thereby enabling valid national and international comparisons of age-standardized prevalence estimates.

### Blood pressure measurement and data cleaning

Blood pressure (BP) was measured by trained healthcare personnel using a validated automated digital sphygmomanometer, following standardized WHO measurement protocols. Three BP readings were obtained for each participant at a single visit, with the participant seated and rested. To minimize measurement variability and potential white-coat effects, the first reading was discarded, and the average of the second and third readings was used for analysis. A predefined data-cleaning protocol was applied to BP measurements. Values coded as “999” were treated as missing. In addition, physiologically implausible values were excluded using the following thresholds: systolic blood pressure values <70 mmHg or >270 mmHg, and diastolic blood pressure values <30 mmHg or >150 mmHg. Participants with missing or implausible systolic or diastolic blood pressure values were excluded from analyses requiring BP measurements.

### Operational definitions

Blood pressure was measured using a standardized protocol in accordance with the WHO STEPwise approach to noncommunicable disease surveillance (STEPS) (17). For each participant, three blood pressure readings were obtained during a single visit while the participant was seated and rested. To minimize measurement variability and potential white-coat effects, the first reading was discarded, and the mean of the second and third readings was used for all analyses (18). Hypertension was operationally defined as having an average systolic blood pressure (SBP) ≥ 140 mmHg and/or diastolic blood pressure (DBP) ≥ 90 mmHg, based on the averaged second and third measurements, or current use of antihypertensive medication, regardless of measured blood pressure values at the time of the survey. This definition is consistent with World Health Organization (WHO) recommendations and widely accepted international and American Heart Association–endorsed epidemiologic standards, allowing comparability with national and global population-based studies (19, 20).

Treatment of hypertension was defined as self-reported current use of antihypertensive medication at the time of the interview among individuals meeting the hypertension definition (11). Information on non-pharmacological management, such as lifestyle modification alone (e.g., dietary changes, physical activity, or salt reduction without medication), was not systematically collected in the survey and therefore was not classified as treatment. Awareness of hypertension was defined as self-reported prior diagnosis of hypertension by a healthcare professional (physician or other qualified health provider) among participants who met the study's measured definition of hypertension (21). Participants with elevated measured blood pressure who reported no prior diagnosis were classified as unaware of their hypertensive status.

These operational definitions are consistent with those used in large national health surveys and global hypertension surveillance studies, ensuring methodological rigor and facilitating valid comparisons across populations and over time.

### Explanatory variables

The selection of explanatory variables for this analysis was informed by a comprehensive review of the literature on hypertension risk factors (3, 10, 22, 23). At the individual level, we examined demographic characteristics including age (categorized into 11 groups from 18 to 34 to ≥80 years), sex, and marital status (never married, currently married, separated/divorced, widowed). Key clinical and anthropometric factors included body mass index (BMI), categorized using WHO classifications as underweight (<18.5 kg/m^2^), normal (18.5–24.9 kg/m^2^), overweight (25.0–29.9 kg/m^2^), and obese (≥30.0 kg/m^2^), along with self-reported comorbidities (diabetes, dyslipidemia, kidney disease) and measured cholesterol levels (desirable: <5.18 mmol/L; borderline high: 5.18–6.18 mmol/L; high: >6.18 mmol/L).

Behavioral factors such as smoking history (ever smoked: yes/no) and physical activity. Physical activity levels were assessed using the Global Physical Activity Questionnaire (GPAQ) Analysis Guide (24). In this survey, physical activity was captured through questions on vigorous exercise, moderate exercise, and walking. However, specific domains such as occupational, recreational, or transport-related activities were not distinguished. To align with the GPAQ framework, these components were matched and labeled with corresponding GPAQ analysis codes, allowing classification of individuals based on physical activity frequency (<3 times/week, 3–5 times/week, or >5 times/week). Socioeconomic status was represented by educational attainment (less than secondary school, secondary school or equivalent, university degree or postgraduate).

### Statistical analysis

All statistical analyses were conducted using R version 4.3.3 (R Foundation for Statistical Computing, Vienna, Austria). Analyses accounted for the complex multistage sampling design of the KSAWHS 2019 by incorporating sampling weights, primary sampling units, and stratification variables to ensure nationally representative estimates. Crude prevalence of hypertension was estimated overall and stratified by age and sex among adults aged 18 years and older. Age-standardized prevalence estimates were calculated using the direct standardization method, with the WHO World Standard Population (2000–2025) as the reference population, applying standard 5-year age categories (15–19, 20–24, …, ≥80 years) (25). Participants aged 15–17 years were included only for age-standardization purposes and were excluded from all other analyses.

Differences in sociodemographic, clinical, behavioral, and biochemical characteristics by hypertension status were assessed using weighted chi-square tests for categorical variables and Wilcoxon rank-sum tests for continuous variables, as appropriate. Associations between hypertension and explanatory variables were examined using Poisson regression models with robust variance estimators, with results presented as prevalence ratios (PRs) and 95% confidence intervals (26). This modeling approach was selected to provide unbiased and interpretable estimates for a common outcome and to avoid overestimation associated with odds ratios from logistic regression. Variables showing statistically significant associations in bivariate analyses were included in the multivariable models. All regression models incorporated survey design features and were evaluated for overall model fit. Statistical significance was defined as a two-sided *p*-value <0.05.

## Results

### Characteristics of the study population

A total of 8,266 adults aged ≥18 years were included in the analysis. Of these, 1,049 participants (12.69%) met the study definition of hypertension based on measured blood pressure and/or current antihypertensive medication use. Table 1 summarizes the sociodemographic, clinical, behavioral, and biochemical characteristics of the study population stratified by hypertension status. Participants with hypertension were significantly older than those without hypertension (mean age: 47.1 ± 16.1 years vs. 35.1 ± 11.7 years, respectively; *p* < 0.001). The prevalence of hypertension increased markedly with age (*p* < 0.001), rising from 6.2% among individuals aged 18–34 years to 54.8% among those aged ≥80 years. Hypertension prevalence differed significantly by sex, with a higher prevalence observed among males (14.1%) compared with females (11.2%) (*p* < 0.001). Marital status was also significantly associated with hypertension (*p* < 0.001); the highest prevalence was observed among widowed individuals (34.0%), followed by those who were separated or divorced (15.7%), and those who were currently married (12.8%), while never-married individuals had the lowest prevalence (7.6%).

**Table 1:** Characteristics of the study population aged ≥18 years, stratified by measured hypertension status.

Variable	Overall (*N* = 8,266)	Measured hypertension status		*p*-value
		No Hypertension (*N* = 7,217; 87.31%) Row %	Hypertension (*N* = 1,049; 12.69%) Row %	
Age, years	36.6 (13)	35.1 (11.7)	47.1 (16.1)	<0.001*
Age category				
18–34	4,429 (53.6%)	4,154 (93.8%)	275 (6.2%)	<0.001*
35–39	1,260 (15.2%)	1,146 (91.0%)	114 (9.0%)	
40–44	774 (9.4%)	674 (87.1%)	100 (12.9%)	
45–49	535 (6.5%)	419 (78.3%)	116 (21.7%)	
50–54	409 (4.9%)	308 (75.3%)	101 (24.7%)	
55–59	293 (3.5%)	200 (68.3%)	93 (31.7%)	
60–64	207 (2.5%)	128 (62.3%)	79 (37.7%)	
65–69	146 (1.8%)	79 (54.1%)	67 (45.9%)	
70–74	88 (1.1%)	47 (52.3%)	41 (47.7%)	
75–79	63 (0.8%)	34 (54.0%)	29 (46.0%)	
≥80	62 (0.8%)	28 (45.2%)	34 (54.8%)	
Sex				
Male	4,416 (53.4%)	3,797 (85.9%)	619 (14.1%)	<0.001*
Female	3,850 (46.6%)	3,420 (88.8%)	430 (11.2%)	
Marital status				
Never married	1,608 (19.5%)	1,485 (92.4%)	123 (7.6%)	<0.001*
Currently married	6,065 (73.4%)	5,290 (87.2%)	775 (12.8%)	
Separated/divorced	281 (3.4%)	238 (84.3%)	43 (15.7%)	
Widowed	312 (3.8%)	204 (66.0%)	108 (34.0%)	
Education				
Less than secondary school	1,065 (12.9%)	772 (72.5%)	293 (27.5%)	<0.001*
Secondary school or equivalent	3,951 (47.8%)	3,490 (88.1%)	461 (11.9%)	
University degree or postgraduate	3,245 (39.3%)	2,953 (90.9%)	292 (9.1%)	
BMI category				
Underweight	260 (3.2%)	233 (88.8%)	27 (11.2%)	<0.001*
Normal	3,043 (37.9%)	2,788 (88.7%)	255 (11.3%)	
Overweight	3,122 (38.8%)	2,768 (87.1%)	354 (12.9%)	
Obese	1,612 (20.1%)	1,287 (79.7%)	325 (20.3%)	
Ever smoked				
Yes	1,136 (14.0%)	960 (84.5%)	176 (15.5%)	0.003*
No	6,986 (86.0%)	6,131 (88.4%)	855 (11.6%)	
Nationality				
Saudi	7,261 (87.8%)	6,344 (87.9%)	917 (12.1%)	0.516
Non-Saudi	1,005 (12.2%)	873 (86.7%)	132 (13.3%)	
Self-reported diabetes				
Yes	695 (8.4%)	465 (67.2%)	230 (32.8%)	<0.001*
No	7,571 (91.6%)	6,752 (89.1%)	819 (10.9%)	
Self-reported dyslipidemia				
Yes	584 (7.1%)	391 (66.1%)	193 (33.9%)	<0.001*
No	7,682 (92.9%)	6,826 (88.9%)	856 (11.1%)	
Self-reported kidney disease				
Yes	27 (0.3%)	11 (40.7%)	16 (59.3%)	<0.001*
No	8,239 (99.7%)	7,206 (87.7%)	1,033 (12.3%)	
Cholesterol level				
Desirable (<5.18 mmol/L)	5,160 (73.5%)	4,640 (89.9%)	520 (10.1%)	<0.001*
Borderline high (5.18–6.18 mmol/L)	1,117 (15.9%)	943 (84.4%)	174 (15.6%)	
High (>6.18 mmol/L)	745 (10.6%)	624 (83.8%)	121 (16.2%)	
Physical activity				
<3 times/week	4,452 (53.9%)	3,745 (84.1%)	707 (15.9%)	<0.001*
3–5 times/week	2,247 (27.2%)	2,063 (91.8%)	184 (8.2%)	
>5 times/week	1,301 (15.7%)	1,193 (91.7%)	108 (8.3%)	
Don't know	266 (3.2%)	216 (81.2%)	50 (18.8%)	

*p*-values were calculated using the Wilcoxon rank-sum test for continuous variables and Pearson's chi-squared test or Fisher's exact test for categorical variables, as appropriate.

* Statistical significance was defined as *p* < 0.05.

Educational attainment showed a strong inverse association with hypertension (*p* < 0.001). Participants with less than secondary education had the highest prevalence (27.5%), compared with those who completed secondary education (11.9%) and those with a university degree or postgraduate education (9.1%). A clear gradient was observed across body mass index (BMI) categories (*p* < 0.001). Hypertension prevalence was highest among obese individuals (20.3%), followed by those who were overweight (12.9%), and lowest among participants with normal weight (11.3%). Underweight participants had a prevalence of 11.2%.

Behavioral and clinical risk factors were also significantly associated with hypertension. Participants who had ever smoked had a higher prevalence of hypertension compared with never-smokers (15.5% vs. 11.6%, *p* = 0.003). Hypertension prevalence was substantially higher among individuals with self-reported diabetes (32.8%), self-reported dyslipidemia (33.9%), and self-reported kidney disease (59.3%), compared with those without these conditions (all *p* < 0.001). Total cholesterol level was significantly associated with hypertension (*p* < 0.001), with prevalence increasing from 10.1% among participants with desirable cholesterol levels, to 15.6% among those with borderline high levels, and 16.2% among those with high cholesterol.

Physical activity frequency was also significantly associated with hypertension (*p* < 0.001). Participants reporting physical activity less than three times per week had a higher prevalence of hypertension (15.9%) compared with those reporting three to five times per week (8.2%) or more than five times per week (8.3%). Participants who reported not knowing their activity frequency had the highest prevalence (18.8%). Nationality was not significantly associated with hypertension prevalence (*p* = 0.516).

### Prevalence and age standardization

 Table 2 presents crude and age-standardized prevalence estimates of hypertension by age group. Among individuals aged 15 years older, the crude prevalence of hypertension was 12.32%. After standardization to the WHO World Standard Population (2000–2025), the age-standardized prevalence increased to 17.32%, reflecting the relatively young age structure of the Saudi population and the strong age dependence of hypertension prevalence. Among adults aged 30–79 years, the crude prevalence of hypertension was 19.41%, while the corresponding age-standardized prevalence was 22.26%, further underscoring the steep increase in hypertension prevalence with advancing age. Age-standardized estimates were calculated using individuals aged 15–17 years solely for standardization purposes, whereas all other analyses were restricted to adults aged 18 years and older.

**Table 2:** Crude age-specific and age-standardized prevalence of hypertension using the WHO world standard population (2000–2025) as reference.

Age group	Total population	Hypertensive count	Crude age-specific prevalence % (95% CI)	WHO world standard as a reference population	Weighted prevalence % (95% CI)
15–19	611	30	4.91 (3.2–6.62)	8.47	0.56 (0.36–0.75)
20–24	983	58	5.9 (4.43–7.37)	8.22	0.66 (0.49–0.82)
25–29	1,489	80	5.37 (4.23–6.52)	7.93	0.58 (0.45–0.70)
30–34	1,698	120	7.07 (5.85–8.29)	7.61	0.73 (0.60–0.85)
35–39	1,260	114	9.05 (7.46–10.63)	7.15	0.88 (0.72–1.03)
40–44	774	100	12.92 (10.56–15.28)	6.59	1.15 (0.94–1.36)
45–49	535	116	21.68 (18.19–25.17)	6.04	1.77 (1.49–2.06)
50–54	409	101	24.69 (20.52–28.87)	5.37	1.80 (1.49–2.10)
55–59	293	93	31.74 (26.41–37.07)	4.55	1.96 (1.63–2.28)
60–64	207	79	38.16 (31.55–44.78)	3.72	1.92 (1.59–2.26)
65–69	146	67	45.89 (37.81–53.97)	2.96	1.84 (1.52–2.16)
70–74	88	41	46.59 (36.17–57.01)	2.21	1.39 (1.08–1.71)
75–79	63	29	46.03 (33.72–58.34)	1.52	0.95 (0.69–1.20)
80–84	37	18	48.65 (32.54–64.75)	0.91	0.60 (0.40–0.80)
85+	25	16	64 (45.18–82.82)	0.63	0.55 (0.39–0.71)
Total	8,618	1,062	12.32 (11.63–13.02)	73.85	____________
Crude prevalence	12.32%			________________________	
Age-standardized prevalence				17.32%	

### Regional variation in hypertension prevalence

**Figure 2:**
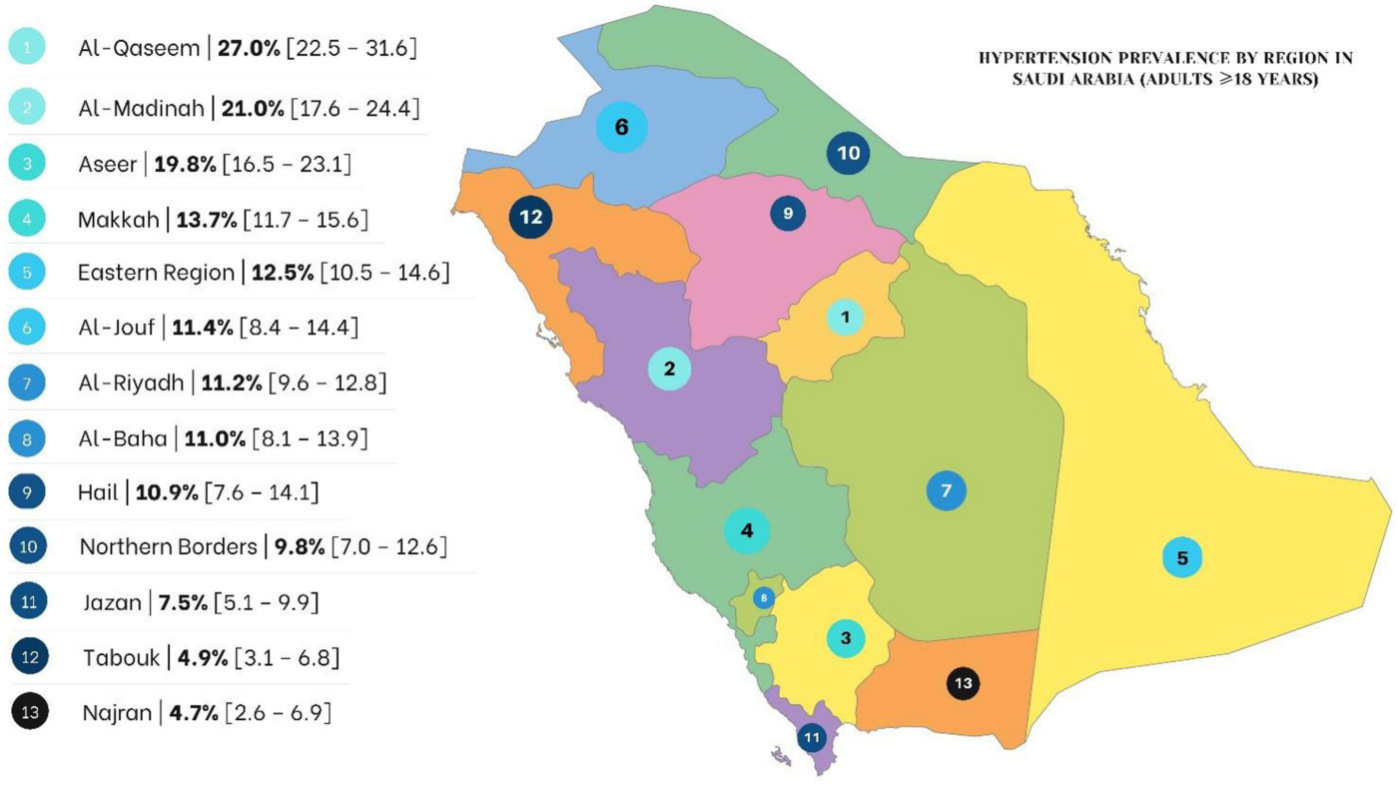
Prevalence of hypertension across different regions in Saudi Arabia. Crude prevalence (%) of measured hypertension among adults aged ≥18 years across the 13 administrative regions of Saudi Arabia. Hypertension was defined as systolic blood pressure ≥140 mmHg and/or diastolic blood pressure ≥90 mmHg, or current use of antihypertensive medication. Regional estimates are weighted and should be interpreted with caution due to variation in regional sample sizes.

 Figure 2 shows the crude prevalence of hypertension across the 13 administrative regions of Saudi Arabia among adults aged 18 years and older. Substantial regional variation was observed. The highest prevalence estimates were recorded in Al-Qaseem (27.0%), followed by Al-Madinah (21.0%) and Aseer (19.8%). In contrast, the lowest prevalence estimates were observed in Najran (4.7%) and Tabuk (4.9%). Intermediate prevalence levels were observed in Makkah (13.7%), the Eastern Region (12.5%), Al-Jouf (11.4%), Al-Riyadh (11.2%), Al-Baha (11.0%), Hail (10.9%), and the Northern Borders (9.8%). These regional estimates should be interpreted with caution, as sample sizes varied across regions and some regions had relatively small numbers of participants.

### Hypertension treatment coverage

Table 3 presents antihypertensive treatment coverage among adults with hypertension, stratified by age group and sex. Among adults aged 30–79 years, 47.96% of individuals with hypertension reported current use of antihypertensive medication. Treatment coverage differed substantially by sex in this age group, with higher coverage observed among females (57.0%) compared with males (41.4%). After age standardization to the WHO World Standard Population, treatment coverage among adults aged 30–79 years was 43.97%. Among adults aged 80 years and older, treatment coverage was markedly higher. Overall, 78.2% of hypertensive individuals in this age group reported receiving antihypertensive treatment. Coverage was slightly higher among females (81.2%) than males (76.7%). The age-standardized treatment coverage for adults aged 80 years and older was 78.64%.

**Table 3:** Coverage of antihypertensive treatment among adults with hypertension, by age group and sex.

Sex	Numerator	Denominator	Coverage of treatment (%)	Age-standardized treatment coverage according to WHO world standard age as reference (%)
For age group (30–79)				
Male	260	628	41.4	________
Female	262	460	57	________
Total	522	1,088	47.96	43.97
For age group (80 and above)				
Male	23	30	76.7	________
Female	13	16	81.2	________
Total	36	46	78.2	78.64

### Awareness of hypertension status

**Figure 3:**
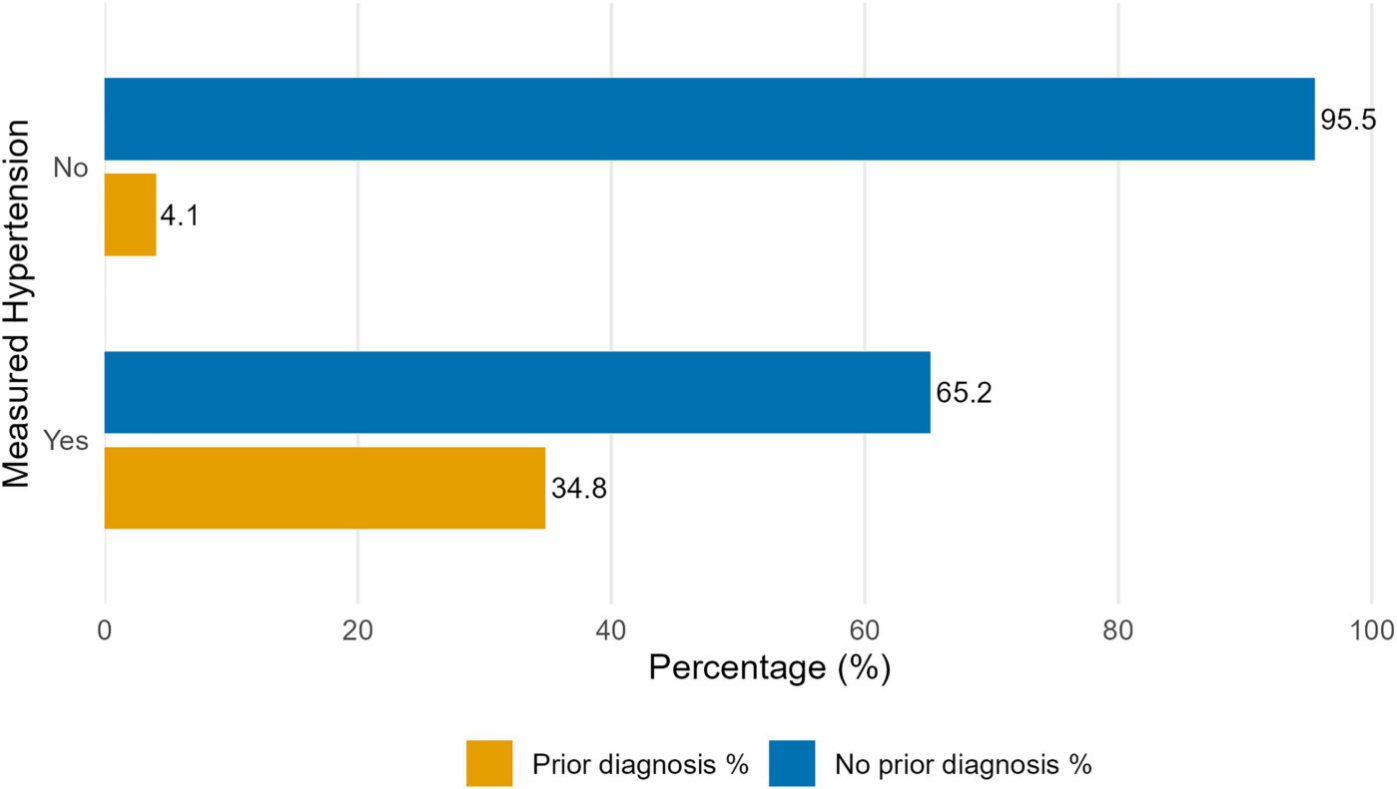
Awareness of hypertension among participants based on blood pressure measurement. Distribution of self-reported prior diagnosis of hypertension by measured hypertension status among adults aged 18 years. Self-reported hypertension refers to a prior diagnosis made by a healthcare professional. Percentages represent survey-weighted proportions within each measured hypertension category. Indicating the proportion of individuals who reported a previous diagnosis among those with and without measured hypertension at the time of survey.

Figure 3 illustrates the concordance between measured hypertension and self-reported prior diagnosis of hypertension among adults aged 18 years and older. Among participants without measured hypertension, 95.9% reported no prior diagnosis of hypertension, while 4.1% reported a history of hypertension despite having normal blood pressure measurements at the time of the survey. Among participants with measured hypertension, only 34.8% reported having been previously diagnosed with hypertension by a healthcare professional, whereas 65.2% were unaware of their hypertensive status.

### Factors associated with hypertension

Table 4 summarizes the results of multivariable Poisson regression models with robust variance estimates examining factors associated with hypertension among adults aged 18 years and older. Compared with individuals aged 18–34 years, the prevalence of hypertension increased progressively with age, reaching an adjusted prevalence ratio (PR) of 8.09 (95% CI: 5.64–11.27) among those aged 80 years and older. Male sex was associated with a higher prevalence of hypertension compared with female sex (PR = 1.22, 95% CI: 1.09–1.36). Educational attainment was inversely associated with hypertension, with individuals who completed secondary education or higher exhibiting lower prevalence compared with those with less than secondary education.

**Table 4:** Factors associated with hypertension among adults aged ≥18 years using Poisson regression with robust variance estimates.

Factor	Prevalence ratio (95% CI)
Age	
18–34	Reference
35–39	1.64 (1.33–2.01)
40–44	2.70 (2.22–3.29)
45–49	3.41 (2.79–4.18)
50–54	3.96 (3.22–4.88)
55–59	4.26 (3.45–5.27)
60–64	5.28 (4.25–6.56)
65–69	8.37 (6.41–10.79)
70–74	7.66 (5.55–10.34)
75–79	7.01 (4.61–10.22)
≥80	8.09 (5.64–11.27)
Sex	
Female	Reference
Male	1.22 (1.09–1.36)
Marital status	
Never married	Reference
Currently married	0.91 (0.74–1.10)
Separated/divorced	1.21 (0.90–1.61)
Widowed	1.26 (0.99–1.61)
Education	
Less than secondary school	Reference
Secondary school or equivalent	0.87 (0.77–0.99)
University degree or postgraduate	0.87 (0.75–1.00)
BMI	
Normal	Reference
Underweight	1.21 (0.86–1.69)
Overweight	1.19 (1.04–1.35)
Obese	1.72 (1.50–1.97)
Ever smoked	
No	Reference
Yes	1.19 (1.05–1.36)
Self-reported diabetes	
No	Reference
Yes	1.34 (1.20–1.50)
Self-reported dyslipidemia	
No	Reference
Yes	1.57 (1.40–1.75)
Self-reported kidney disease	
No	Reference
Yes	2.05 (1.55–2.70)
Cholesterol	
Desirable	Reference
Borderline high (5.18–6.18 mmol/L)	1.24 (0.97–1.60)
High (>6.18 mmol/L)	1.12 (0.84–1.48)
Physical activity	
<3 times/week	Reference
3–5 times/week	0.94 (0.72–1.23)
>5 times/week	1.02 (0.78–1.34)
Don't know	1.22 (0.85–1.75)

Body mass index showed a strong independent association with hypertension. Obesity was associated with a substantially higher prevalence of hypertension (PR = 1.72, 95% CI: 1.50–1.97), while overweight status was also associated with increased prevalence (PR = 1.19). Several behavioral and clinical comorbidities were significantly associated with hypertension, including ever smoking (PR = 1.19), diabetes (PR = 1.34), dyslipidemia (PR = 1.57), and kidney disease (PR = 2.05).

## Discussion

This study provides a comprehensive overview of the prevalence, treatment patterns, and factors associated with hypertension among adults in Saudi Arabia. Our findings show that hypertension remains a significant public health concern, affecting approximately 12.69% of adults, with an age-standardized prevalence of 16.5%. These rates are broadly consistent with previous national estimates, although they appear slightly higher than those reported by Alenazi et al., who found a prevalence of 9.2% based on data from the 2017 national health survey (27). This difference may be due to variations in case definition. Alenazi et al. included only individuals with a confirmed diagnosis made by a specialist. Their estimate was based on self-reported, previously diagnosed cases. In contrast, our study used direct blood pressure measurements. This approach allowed us to identify both diagnosed and undiagnosed hypertension, leading to a higher and likely more accurate estimate.

Compared to other countries in the region, the prevalence of hypertension in Saudi Arabia is relatively low. In our study, the age-standardized prevalence was 17.2%. This is lower than reported rates in several Arab countries. For example, hypertension prevalence is 33.8% in Jordan, 29.3% in Lebanon, and 41.5% in Oman (28–30). The United Arab Emirates also reports a higher rate of 24% (31). These regional differences may reflect variations in healthcare access, awareness, and population structure. Globally, Saudi Arabia also reports lower hypertension rates. The worldwide average is estimated at around 32% (32). High-income countries show similar or even higher levels. For instance, the prevalence is 33% in Australia, 34% in Canada, and as high as 59% in Finland. In the United States, 32.9% of adults have hypertension (33, 34). These differences likely reflect aging populations, lifestyle factors, and healthcare systems. When comparing countries, age structure and access to care are key influences on national hypertension estimates (35, 36). It is also important to interpret these cross-national comparisons with caution, as differences in hypertension prevalence across countries may be influenced by methodological heterogeneity, including variations in age ranges, sampling strategies, blood pressure measurement protocols, and case definitions. These factors, in addition to true epidemiological differences, may partially explain the higher prevalence reported in neighboring countries compared with Saudi Arabia.

As observed in many other contexts, age was a dominant determinant of hypertension in our study. The prevalence increased markedly with advancing age, reaching over 50% among individuals aged 80 years and above. This aligns with findings from both Alenazi et al. and Nasser et al., who similarly documented a strong age gradient in hypertension prevalence (27, 37). Gender differences were also evident, with men exhibiting a higher prevalence than women, consistent with previous studies in Saudi Arabia and internationally (27, 38, 39).

Educational attainment emerged as a protective factor against hypertension, with individuals attaining secondary or higher education having lower prevalence rates. This pattern echoes findings from Nasser et al., suggesting that education may contribute to better health literacy, healthier lifestyles, or greater access to healthcare resources (37). Moreover, sociodemographic factors such as marital status were associated with hypertension, with widowed individuals demonstrating the highest prevalence rates, potentially reflecting the compounded effects of age, stress, and social isolation (35, 40).

Several clinical and behavioral risk factors were independently associated with hypertension in our cohort. Obesity was a particularly strong predictor, consistent with global and local studies, including those by Nasser et al. and Alshammari et al. (37, 38). Additionally, smoking, diabetes, dyslipidemia, and kidney disease were all significantly linked to higher hypertension prevalence (41).

Notably, while elevated cholesterol levels showed an association with hypertension in unadjusted analyses, this association was attenuated after controlling other factors, suggesting that cholesterol may act through mediating pathways involving other cardiometabolic risks (42). Interestingly, physical activity showed a non-significant but protective trend in multivariable analysis, highlighting the potential benefit of promoting active lifestyles as a preventive strategy (43).

Geographic variations in hypertension prevalence were striking. Al-Qaseem, Al-Madinah, and Aseer regions reported the highest rates, whereas Najran and Tabuk had the lowest. These patterns align with previous reports of regional heterogeneity in hypertension across Saudi Arabia (27), and may reflect differences in socioeconomic status, healthcare access, lifestyle behaviors, and genetic predispositions (3, 11, 44).

Despite the relatively high prevalence of hypertension, a particularly concerning finding of this study is the low level of hypertension awareness, with only 34.8% of individuals with objectively measured hypertension reporting a prior diagnosis. These findings parallel global concerns highlighted by Mills et al., who noted that awareness, treatment, and control rates for hypertension remain suboptimal, particularly in low- and middle-income countries (26, 41). This level of awareness is comparable to, or lower than, estimates reported in several low- and middle-income countries and underscores a critical gap in the hypertension care cascade (26, 41). Several factors may contribute to this low awareness. Hypertension is frequently asymptomatic, leading many individuals to remain undiagnosed unless actively screened. In Saudi Arabia, blood pressure measurement is often opportunistic rather than systematic, particularly among younger adults and men, who may have limited engagement with preventive healthcare services. Additionally, suboptimal utilization of primary care for routine health assessments and limited public awareness of hypertension as a silent risk factor may delay diagnosis. These findings highlight the urgent need for population-level screening initiatives, integration of routine blood pressure measurement into primary healthcare encounters, and targeted public health campaigns aimed at improving hypertension awareness and early detection.

Treatment patterns further revealed low medication use among young adults and moderate use among older adults, with women more likely than men to report being on treatment. This pattern aligns with findings from a U.S. study, which reported that the young adults diagnosed with hypertension had the lowest prevalence of antihypertensive medication use (49%), compared to 73% among middle-aged and 80% among older adults (45). The U.S. study further highlighted that among young adults, males, individuals with mild hypertension, and White patients experienced slower rates of medication initiation, while those covered by Medicaid and those with more frequent clinic visits had faster initiation rates (33). These findings suggest that both demographic factors and healthcare access influence treatment uptake and emphasize the need for targeted interventions to improve early hypertension management, particularly among younger populations (45, 46).

Despite the lower overall prevalence in Saudi Arabia relative to some of its regional neighbors and high-income countries, the high age-standardized rate of hypertension in this study suggests that the condition is still a major concern, particularly as the population ages and adopts more sedentary lifestyles. Addressing hypertension through public health initiatives, early detection, and effective treatment strategies will be crucial in mitigating the long-term impacts of this widespread condition in Saudi Arabia.

## Limitations

This study has several limitations that should be considered when interpreting the findings. First, the cross-sectional design precludes inference of causal relationships between hypertension and associated sociodemographic, behavioral, and clinical factors. The observed associations should therefore be interpreted as correlational. Second, blood pressure was measured during a single visit, which may result in misclassification due to short-term variability and the absence of a confirmatory measurement on a separate occasion. As a result, hypertension prevalence may be overestimated, particularly among individuals with transiently elevated blood pressure or white-coat hypertension.

Third, several key variables, including diabetes, dyslipidemia, kidney disease, smoking history, and physical activity, were based on self-reported information, which is subject to recall and social desirability bias. In particular, cardiometabolic conditions such as diabetes and dyslipidemia may be underreported, especially among individuals with limited access to healthcare or low awareness of their health status. This underreporting may lead to underestimation of their true prevalence and attenuation of associations with hypertension. Fourth, although the survey was nationally representative, sample sizes within some administrative regions were relatively small. This may have reduced the precision of regional prevalence estimates and should be considered when interpreting geographic variations in hypertension burden.

Fifth, the analysis was based on secondary data from an existing national survey, which limited the scope of available variables. Notably, detailed dietary information, including salt intake, fruit and vegetable consumption, and overall dietary patterns, was not collected despite their well-established role in hypertension etiology. The absence of these variables may have resulted in residual confounding and limits the ability to fully assess lifestyle-related determinants of hypertension. Finally, while several of the associations identified in this study are well established in the literature, the unique contribution of this analysis lies in the use of objectively measured blood pressure, nationally representative data, and the simultaneous examination of hypertension prevalence, awareness, treatment coverage, and regional variation within Saudi Arabia using standardized methods. These features provide updated, policy-relevant evidence that complements and extends prior analyses based on self-reported diagnoses or smaller subnational samples.

## Conclusion

This study highlights that hypertension remains a significant health burden among adults in Saudi Arabia, with a prevalence of 12.69%, increasing sharply with age. The most concerning finding of this nationally representative study is the low awareness of hypertension, with only one-third of individuals with objectively measured hypertension reporting a prior diagnosis. This gap substantially limits effective treatment and control, despite Saudi Arabia's moderate overall hypertension prevalence. Hypertension was strongly associated with male sex, older age, obesity, smoking, diabetes, dyslipidemia, and kidney disease, while higher educational attainment was protective. Notable regional variation highlights the need for targeted interventions. Improving hypertension control in Saudi Arabia requires prioritizing systematic and opportunistic blood pressure screening within primary healthcare, in alignment with ongoing Primary Health Care Transformation efforts. Community-based screening programs and context-specific public health education campaigns can further enhance early detection and awareness, particularly among younger adults and men. Collectively, these strategies are critical to reducing the long-term cardiovascular burden of hypertension in Saudi Arabia.

## Data Availability

The original contributions presented in the study are included in the article/Supplementary Material, further inquiries can be directed to the corresponding author/s.
